# Persistent severe muscle pain following mistakenly injected high-dose bee venom: A care-compliant case report

**DOI:** 10.1097/MD.0000000000032180

**Published:** 2022-12-09

**Authors:** Min Cheol Chang

**Affiliations:** a Department of Rehabilitation Medicine, College of Medicine, Yeungnam University, Daegu, Republic of Korea.

**Keywords:** adverse effect, bee venom, lower back pain, pain, toxin

## Abstract

In clinical practice, bee venom injection into acupuncture points is used to relieve various types of musculoskeletal pain. In the current study, we describe a patient who had persistent severe muscle pain caused by mistakenly injected high-dose bee venom.

**Patient concerns:**

A 63-year-old woman mistakenly received an injection of high-dose (2 mL; standard dose, 0.1 mL) bee venom (diluted in saline at a 1:2000 ratio). Immediately after the injection, extreme burning pain developed at the injection site, which persisted for 1 month with a mean pain score of 9 on the numeric rating scale. T1-weighted gadolinium-enhanced axial lumbar magnetic resonance imaging revealed increased intensity in the medial part of the left psoas muscle around the L4-5 intervertebral disc level.

**Diagnosis:**

This finding indicated the presence of inflammation in the left psoas muscle, which was thought to be associated with pain.

**Interventions:**

A buprenorphine transdermal patch (releasing 5 mcg/hours) was applied to alleviate the pain.

**Outcomes:**

Six months after the bee venom injection, the pain completely resolved.

**Lessons:**

Bee venom has a strong toxic effect; therefore, only a minimal dose of diluted bee venom should be administered for musculoskeletal pain control. Special caution is required during bee venom injection to avoid excessive doses of bee venom.

## 1. Introduction

Injection of diluted bee venom is 1 of the most commonly used traditional complementary and alternative therapeutic methods, which has been believed to be effective in the treatment of several diseases, such as rheumatic arthritis, tendinitis, herpes zoster, bursitis, gout, and burns.^[1]^ Additionally, it can be applied to reduce various types of musculoskeletal pain, mainly in East Asian countries.^[2,3]^

Lower back pain is 1 of the most common complaints of patients who visit hospitals or clinics. An individual has a 60 to 85% lifetime chance of experiencing lower back pain.^[4]^ Lower back pain frequently limits daily activities and lowers the quality of life.^[5]^ Additionally, it is a leading cause of absence from work worldwide.^[5]^ Several therapeutic tools, such as oral medications, physical therapies, and injection procedures, are used to control lower back pain.^[6,7]^ Bee venom injection into acupuncture points has also been used for relieving lower back pain.^[8,9]^

However, bee venom injections can cause a few complications. First, in sensitized patients, it may cause allergic reactions, including systemic or anaphylactic reactions, which can be life-threatening.^[10]^ An epidemiological investigation reported that approximately 3% of adults had a systemic reaction to the bee sting.^[10]^ Second, even though venom has been reported to have anti-nociceptive and anti-inflammatory effects, it can cause pain and inflammation in the injected areas as it is harmful to the human body.^[1]^ Many studies have demonstrated the strong toxicological effects of bee venom and its major polypeptide (melittin) on pain-producing and pain-conducting systems.^[1]^ Due to allergic reactions and the strong toxicological effects of bee venom, only a minimal dose of highly diluted bee venom is injected into acupuncture points.

Here, we describe a patient who had persistent severe muscle pain caused by mistakenly injected high-dose bee venom during the treatment of lower back pain.

## 2. Case report

A 63-year-old woman presented to the pain clinic of a university hospital with severe left lower back pain. Owing to severe pain, the patient was unable to stand or walk. The patient had no specific medical history or psychological disorder. Her left lower back pain started 1 month prior to the mistakenly injected high-dose bee venom in the local clinic. The physician at the local clinic conducted a trigger point injection (TPI) into the left psoas muscle. When the physician injected the TPI solution, the patient complained of extreme burning pain at the injection site. The physician realized that he had injected 2 mL bee venom (standard dose, 0.1 mL) diluted in saline at a 1:2000 ratio instead of the TPI solution of normal saline and 2% lidocaine.

**Figure 1.**
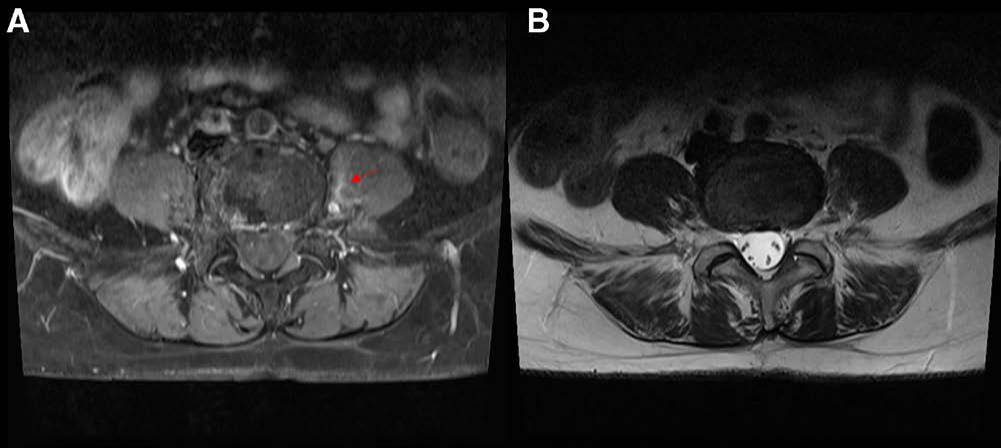
(A) T1-weighted gadolinium-enhanced axial MRI shows increased intensity in left psoas muscle (red arrow) (B) Conventional T2-weighted MRI shows no abnormal finding in left psoas muscle. MRI, magnetic resonance imaging. MRI = magnetic resonance imaging.

Physical examination revealed no motor weakness or sensory deficits in the lower extremities. Knee and ankle jerks were normal bilaterally. The patient’s left lower back pain aggravated during passive or active flexion or extension of her left hip joint. Her pain was burning and sharp in nature, with a pain score of 9 on the numeric rating scale (NRS). An electrophysiological study (nerve conduction study and electromyography) performed 1 month after the bee venom injection showed normal findings. T1-weighted gadolinium-enhanced axial lumbar magnetic resonance imaging (MRI) revealed increased intensity in the medial part of the left psoas muscle around the L4-5 intervertebral disc level (Fig. 1A). Conventional T2- and T1-weighted MRI showed no abnormal findings in the left psoas muscle (Fig. 1B). Lumbar MRI findings indicated the presence of inflammation in the left psoas muscle, which was considered to be induced by mistakenly injected high-dose bee venom. Inflammation of the left psoas muscle appeared to cause of patient’s pain. For pain management, the patient refused to receive oral medication for pain control, and only a buprenorphine transdermal patch (releasing 5 mcg/h) was applied. Two months after the bee venom injection, her pain reduced from an NRS score of 9 to an NRS score of 7. Four months after the bee venom injection, the NRS score was 3. Six months after the bee venom injection, her pain completely disappeared, and no physical impairment was reported.

The study was approved by the local institutional review board of Yeungnam University hospital. Written informed consent was obtained from the patient and the patient’s legal guardian for publication of this case report and accompanying images.

## 3. Discussion

Here, we report a patient who developed severe left lower back pain after injection of bee venom (diluted at a 1:2000 ratio) with a dose of 20 times more than the standard dose into the left psoas muscle. The pain caused by the bee venom injection persisted for several months.

The main and most toxic compound in bee venom is melittin, a major polypeptide consisting of 50 to 60% of the whole bee venom.^[11]^ It induces pain associated with bee stings through direct and indirect actions on primary nociceptor cells.^[12]^ Its direct action is the activation of the thermal nociceptor transient receptor potential vanilloid 1 through the phospholipase A2 cascade pathway, causing sensitization of the primary nociceptor.^[13,14]^ Additionally, melittin indirectly induces the release of pain-inducing substances such as adenosine triphosphate, 5-hydroxytryptamine, H^+^, histamine, and bradykinin.^[13,14]^ Furthermore, mast cell-degranulating peptide is another important peptide in bee venom that induces histamine release from mast cells and plays a central role in the inflammatory process.^[15]^ Hyaluronidase is an enzyme found in bee venom that induces the fast spread of the toxin.^[16]^ The above-mentioned inflammation- and pain-producing materials in the venom seem to induce inflammation or tissue damage in the patient’s psoas muscle and cause severe pain.

Bee venom has promising anti-inflammatory and immunomodulatory characteristics.^[17]^ Therefore, it is widely used to control inflammation and alleviate musculoskeletal pain in Asia, Eastern Europe, and South America.^[17]^ Several clinical trials have been conducted to evaluate the effectiveness of bee venom for managing lower back pain and have demonstrated its positive therapeutic effect.^[8,9,18]^ As bee venom has a strong toxic effect, only a minimal dose of diluted bee venom is allowed in clinical practice. Bee venom is colorless and can be confused with other injection materials such as normal saline or dexamethasone. When it is injected with a dose beyond the allowed range, severe pain, as in our case, or a life-threatening systemic reaction can occur. Therefore, special caution is required during pain control treatment using bee venom.

In conclusion, we described a case of severe pain around the injection site after mistakenly injecting 2 mL of bee venom (diluted at a 1:2000 ratio) for the lower back pain treatment. To the best of our knowledge, this is the first report of a side effect following bee venom injection at a dose exceeding the permitted dose range.

## Author contributions

**Conceptualization:** Min Cheol Chang.

**Data curation:** Min Cheol Chang.

**Formal analysis:** Min Cheol Chang.

**Investigation:** Min Cheol Chang.

**Methodology:** Min Cheol Chang.

**Resources:** Min Cheol Chang.

**Supervision:** Min Cheol Chang.

**Validation:** Min Cheol Chang.

**Visualization:** Min Cheol Chang.

**Writing – original draft:** Min Cheol Chang.

**Writing – review & editing:** Min Cheol Chang.
